# Edible Crabs “Go West”: Migrations and Incubation Cycle of *Cancer pagurus* Revealed by Electronic Tags

**DOI:** 10.1371/journal.pone.0063991

**Published:** 2013-05-29

**Authors:** Ewan Hunter, Derek Eaton, Christie Stewart, Andrew Lawler, Michael T. Smith

**Affiliations:** Centre for Environment, Fisheries and Aquaculture Science, Lowestoft Laboratory, Lowestoft, Suffolk, United Kingdom; University of Sydney, Australia

## Abstract

Crustaceans are key components of marine ecosystems which, like other exploited marine taxa, show seasonable patterns of distribution and activity, with consequences for their availability to capture by targeted fisheries. Despite concerns over the sustainability of crab fisheries worldwide, difficulties in observing crabs’ behaviour over their annual cycles, and the timings and durations of reproduction, remain poorly understood. From the release of 128 mature female edible crabs tagged with electronic data storage tags (DSTs), we demonstrate predominantly westward migration in the English Channel. Eastern Channel crabs migrated further than western Channel crabs, while crabs released outside the Channel showed little or no migration. Individual migrations were punctuated by a 7-month hiatus, when crabs remained stationary, coincident with the main period of crab spawning and egg incubation. Incubation commenced earlier in the west, from late October onwards, and brooding locations, determined using tidal geolocation, occurred throughout the species range. With an overall return rate of 34%, our results demonstrate that previous reluctance to tag crabs with relatively high-cost DSTs for fear of loss following moulting is unfounded, and that DSTs can generate precise information with regards life-history metrics that would be unachievable using other conventional means.

## Introduction

Routinely obtaining frequent, repeated and accurate estimates of the location of marine animals and an accompanying description of spatial and temporal behaviour patterns has for many years presented marine scientists with a significant technical challenge [1]. This challenge has been partially addressed in recent years for large to medium-sized marine vertebrates (e.g. [2], [3]) with the rapid development of biologging technologies [4]. Progress with archival tagging in particular has significantly advanced understanding of the spatial structure and population dynamics of fishes, including smaller, sea-bed dwelling species including flatfish (e.g. [5], [6]), gadoids (e.g. [7]–[9]) and elasmobranchs (e.g. [10], [11]), often with application to fisheries management (e.g. [12]–[14]). Even some invertebrate species have been targeted (e.g. [15], [16]). However progress has been slower for smaller, mobile species living at or near the seabed [1].

Obtaining long-term spatial information on Decapod crustaceans such as crabs, often key components in marine ecosystems (e.g. [17], [18]) and subject to extensive and commercially valuable fisheries worldwide [19], [20]), presents a further, unique set of challenges. Most crabs remain in contact with the sea-bed at all times rather than periodically rising into the water column, as may be the case for many demersal fish (e.g. [21]). Furthermore, crabs are adapted to a reptant (“creeping”) lifestyle so it is important that tag attachment does not impede natural behaviours, such as burrowing in sediment and entry to rock crevices. Locally detailed information on habitat use can be gained from acoustic or ultrasonic tracking [22]–[24], but spatial coverage is limited and precision can be adversely affected by seabed features and can be lost altogether when the animal moves into a crevice or burrow. Electromagnetic telemetry also operates at a local scale over short time periods and studies are also limited by the short range of detection and the requirement for cables on the seabed [25]–[27].

There are however, aspects of crab morphometrics that offer some potential advantages for archival tagging. For example, tag weight may be less important than for finfish [28], [29]. This may permit the use of larger tags with bigger, more powerful batteries and hence increased data storage capacity. Indeed, the utility of archival tagging has already been demonstrated in localised studies of the reef-wide movements of spider crabs [30], and in determining habitat choice behaviour in the Dungeness crab, *Cancer magister* [31], [32] in an estuary. However, perhaps the main barrier to the routine use of archival tagging in crustacean studies historically, at least on a broad geographical scale, has been the unit cost of the tags relative to the perceived probability of tag loss due to moulting (i.e. periodic shedding of the exoskeleton as part of the growth process). However, the cost of archival tags has reduced substantially in recent years (from over £1000 per unit 15 years ago to under £300 per unit today, depending on the sensors). By targeting tagging at larger animals (which moult less frequently) early in their inter-moult period, the potential to retrieve large quantities of high quality behaviour data in large-scale tag releases now potentially outweighs the risks associated with tag loss, making the cost of large-scale tagging experiments scientifically and financially more viable.

In U.K. waters, edible crab (*Cancer pagurus*) is one of the most important commercial fisheries, yet there remain several important gaps in our understanding of their biology and ecology. Mark-recapture experiments carried out in the English Channel in the 1970s [33]–[35] indicated long distance movements, particularly by mature females, and predominantly along an east to west axis. However, in the 40 years since this work was completed [33], total landings in the English crab fishery have effectively quadrupled [19], and the average sea surface temperature in the English Channel has risen by approximately 1°C (www.mccip.org.uk).

In the present study, mature female edible crab were tagged with electronic data storage tags and released at selected locations in some of the most intensively fished crab fisheries in U.K. waters. As far as we are aware, this is the first bulk release of DST-tagged crabs over a wide geographical scale which has been solely reliant upon the commercial fishery for tag returns. With no cohesive current picture of stock identity, our aim was to describe adult crab movements, and to quantify the conditions, timing and duration of reproductive behaviour. Understanding how, where and when crabs undergo large scale migrations is the key to successful stock assessment and management and is important in identifying key life stages (e.g. egg incubation) and periods that may be vulnerable to local fishing or other human activities.

## Materials and Methods

### 1. Release of Edible Crabs Tagged with Electronic Data Storage Tags

Between August 2008 and June 2009, 128 pot-trapped, female edible crabs (carapace width 138–228 mm, mean = 178.8±19.0) were tagged with Cefas G5 long-life (2 MB memory capacity) electronic data storage tags (DSTs), with 2 MB memory capacity (CTL Ltd., Lowestoft, U.K.), configured with a 10 bar pressure sensor (reliable to ∼100 m depth). Earlier aquarium trials identified that crevice burrowing by crabs between boulders could abrade unmodified DSTs through to the internal circuitry within periods as short as one month. To improve abrasion resistance, crab DSTs for wild deployment were therefore encased in secondary, lozenge-shaped perspex casings (bevelled along the outer edges) measuring 32×12×16 mm. To maximise high resolution data collection, DSTs were programmed to record pressure at 30 s intervals and temperature at 5 min intervals for the first year at liberty, then both parameters at 5 min intervals thereafter.

Previous mark-recapture studies have suggested that migration by male edible crab is limited [33], [23], therefore to maximise viable return rates, only female crab have been targeted in this study. To minimise the potential impact of moulting on tag loss, only recently moulted (“new shell”) crabs with no obvious external damage were selected for tagging. Individual crabs were double-tagged. First, a uniquely numbered claw tag (coloured plastic cable-ties), was attached to the right-hand cheliped. The carapace was patted dry using absorbent paper and any fouling material on and around the area of attachment was removed. A small dab of superglue was applied centrally to the base of the DST, with fast-setting underwater epoxy resin applied around the basal perimeter. DSTs were then glued dorsally to the posterior carapace. The long axis of the tag was positioned parallel to, but not obstructing the epimeral line, with the tag label on the vertical plane (to avoid removal by abrasion on the top surface). The instant purchase obtained through the superglue counteracts the relatively slow curing time of the resin (approximately 2 h at room temperature). This allowed the resin to set fully with the tag firmly in place, obtaining a lasting bond between tag and crab. DST and tag numbers were checked on release and the position and time recorded using a handheld GPS (Thales “Mobile Mapper”, Thales Navigation Inc., France). The maximum time between capture, tagging and redeployment was approximately 30 min.

**Figure 1:**
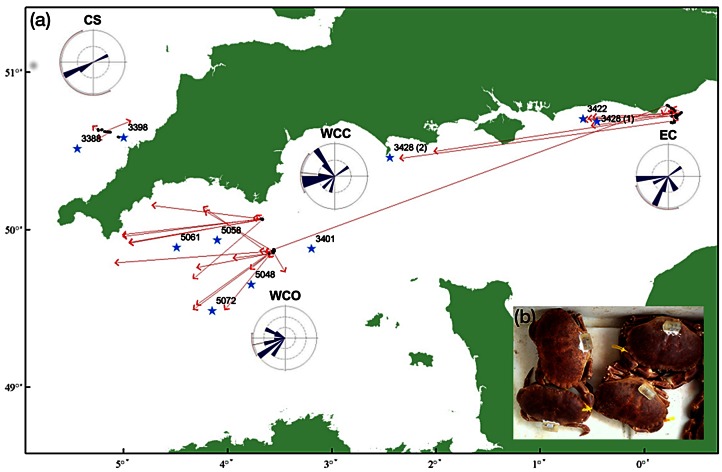
Migration routes and brooding locations of edible crabs *Cancer pagurus* tagged with electronic data storage tags (DSTs). (a) Release and recapture locations, estimated brooding locations (see text), and distance and direction of movement by edible crabs tagged with electronic data storage tags and released in the English Channel and Celtic Sea from August 2008 to June 2009. Rose diagrams illustrate the mean axes (and 95% confidence limits) of migration. Key: Black dots, release locations; Red lines, direction of travel; red arrowheads, recapture locations; Stars, brooding locations of crabs (tag number) estimated using the tidal location method; EC, Eastern Channel; WCC, Western Channel Coastal; WCO, Western Channel Offshore; CS, Celtic Sea. (b) DST-tagged crabs prior to release.

The tagged crabs were released at 4 locations in UK waters (Figure 1, Table 1): Eastern Channel (“EC”); Celtic Sea (“CS”); Western Channel Coastal (“WCC”); and Western Channel Offshore (“WCO”). No specific permissions were required for our tagging work, which was executed in international waters outside of the 12-mile UK territorial limit, and did not, therefore, require authorisation. Note that the edible crab, *Cancer pagurus*, is neither an endangered nor protected species. Although experimentation using live decapods crustaceans is not currently regulated in the UK or European Union, the highest standards of animal welfare were applied throughout our work. All tags were returned through the commercial fishery following a concerted publicity campaign, and the offer of financial rewards (£50) for each returned tag.

**Table 1:** Mark-recapture results.

	Release date	Releases	Recaptures	Tag loss	Recorded days	Total days	Distance	Direction
Eastern Channel (EC)	Aug-08	32	12 (38)	1	1582 (8, 575)	1692	64.2±107.2	207.8° ±61.4°
Celtic Sea (CS)	Oct-08	29	5 (17)	0	514 (37, 254)	713	14.5±9.0	245.1° ±67.8°
Western Channel Coastal(WCC)	Jun-09	30	12 (40)	0	1025 (1, 268)	1171	48.6±43.9	276.6° ±54.9°
Western Channel Offshore(WCO)	Jun-09	37	14 (38)	0	1397 (9, 383)	1965	51.9±50.5	258.2° ±30.9°
Total		128	43 (34)	1	4519	5540		

Summary of release information by area and recapture data (recapture percentage in brackets) for edible crab tagged with electronic data storage tags. Recorded days (minimum and maximum data records in brackets) vs. Total days at liberty = the total number of days at liberty recorded by the electronic tags. Distances in kms ± s.d., directions in degrees ± circular s.d.

### 2. Data Download and Processing

Following return, individual DSTs were downloaded, and pressure data converted into depth. Plots were made of individual depth and temperature experience, and summarized by release on a monthly basis. Temperatures recorded by the tagged crabs were compared with temperatures monitored daily in the eastern Channel at 50.766N 0.300E (“Eastbourne”, Cefas coastal monitoring) and approximately monthly in the western Channel (50.033 N, 4.367 W, “E1” CTD seabed temperature, Western Channel Observatory).

Where crabs appeared to be resting motionless on the sea-bed, we attempted to estimate their position using the tidal location method or “TLM” [36]. This technique estimates geographical location (“geolocation”) based on the time of high water and tidal range, measured by the DST’s depth (pressure) sensor when a tagged-individual remains motionless on the sea-bed over a full tidal cycle (or longer). Tidal ranges were extracted using a wave-fitting algorithm (see [36] for full details). Starting at each successive point in the DST pressure record, the algorithm searched for the best fitting sine-wave, applying a least-squares regression, using data from the following nine-hour period. The period of the model wave-form was constrained so that the half-tidal period could not fall below 4.5 hours or exceed 7.5 hours. The offset of the model was constrained so that the wave-form began with a maximum (or minimum) and continued beyond a minimum (or maximum). The daily best-fitting wave-form was used to calculate the times of high and low water, the tidal range, and to provide an indication of the quality of fit (sum of squares). Unlike recapture positions, which are dependent on recapture by fishers, the geolocations generated by the TLM are independent of the spatial distribution of the fishing fleet [37].

Where possible, incorrect geolocations were eliminated by comparing sea-bed depths (taken from British Admiralty Charts) at the derived geolocations with the actual depths recorded by the tag. Recorded temperatures were compared with averaged sea-surface temperatures (SST, taken from Bundesamt für Seeschiffahrt und Hydrographie, BSH). When depths for derived positions differed by more than ±10 m from the actual recorded sea-bed depth, or temperatures differed by more than ±1°C of the tag-recorded temperature, these positions were eliminated from the analyses. Where the method identified clusters of geolocations rather than individual points, the geographical midpoint was determined as the best fit.

## Results

### 1. Recapture Rates of Tagged Crabs

**Figure 2:**
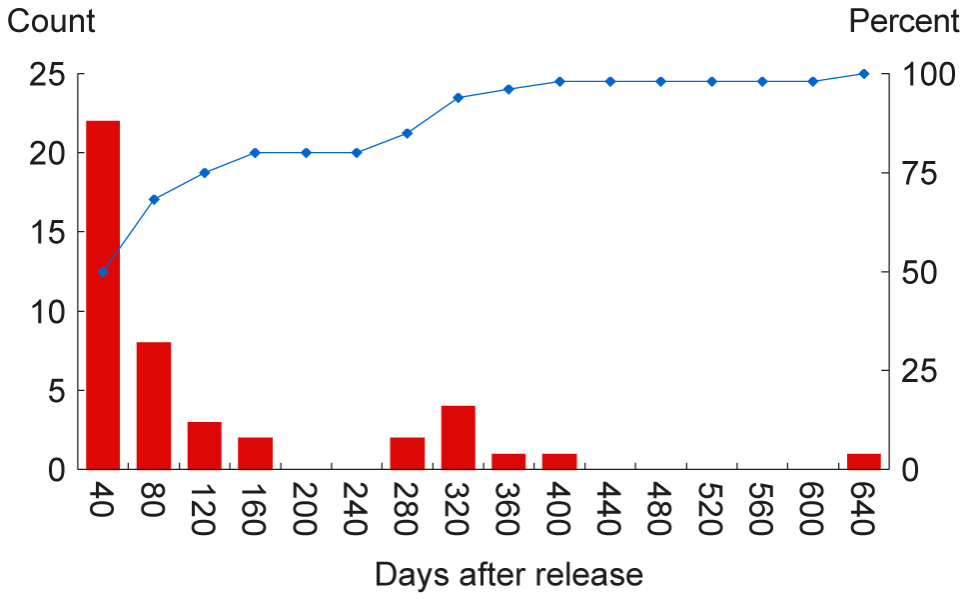
Return rates of tagged crabs. Return rate of edible crabs tagged with electronic data storage tags recaptured per 40 day time interval following release (histogram), and cumulative percentage recapture by time (line).

To date (July 2012), 43 DSTs have been returned (34%, Table 1). Return rates between release sites varied between 17% (CS) and 40% (WCC). Only one individual was missing its DST on recapture (3413, EC). A second individual was recaptured, then immediately re-released once the tag details had been noted (5077, WCO). Individual data records ranged between 8 and 575 days, and 4519 days of high resolution crab behaviour data were captured from 5540 days at liberty (Table 1). Fifty percent of DST recaptures were made during the first 40 days after release (Figure 2). The recapture rate thereafter did not follow a regular diminution pattern, but was seasonally distributed, with zero recaptures over the winter months.

### 2. Distance and Direction Travelled

Tagged crabs were recaptured between 0.7 and 302.4 km from the point of release, from one to 679 days following release (Table 1), having recorded between 1 and 575 day long data records. The direction of movement in all releases followed a predominantly westward axis (Table 1). This was pronounced in the English Channel (Figure 1), where mean crab displacement was 64.2 km along an average vector of 207.8° in the EC (p = 0.05), and 48.6 km and 51.9 km along vectors of 276.6° (p = 0.01) and 258.2° (p>0.001) in WCC and WCO respectively (Table 1). CS crabs moved on average just 14.5 km from the point of release, and although this movement was also predominantly westwards (245.1°), this result was non-significant (p = 0.4). No long-distance migrations (≥15 km) followed a west-to-east axis (Figure 1).

### 3. Physical Data Recorded by the Tags

**Figure 3:**
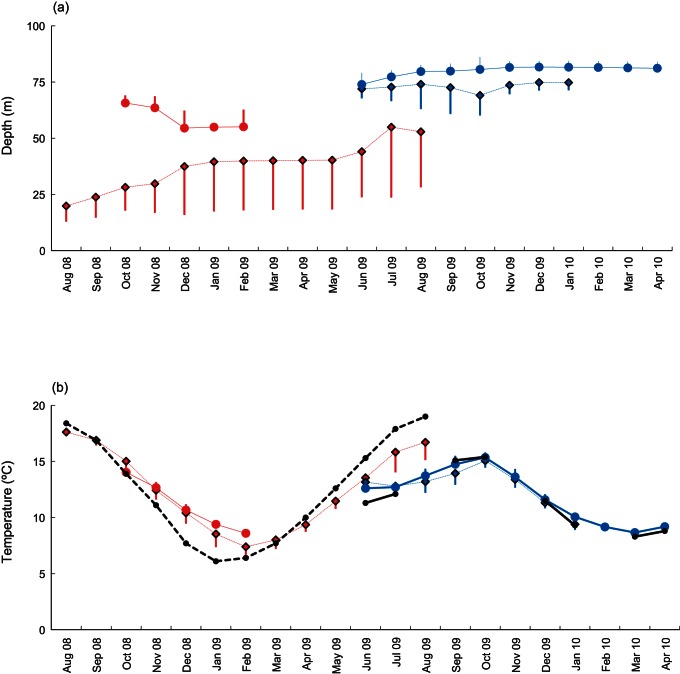
Average depth and temperature experience of edible crab. Average monthly (a) depths and (b) temperatures (± s.e) experienced by DST-tagged crabs released in the English Channel and Celtic Sea. Black lines (b only) indicate average monthly temperatures in the eastern Channel (broken line) and approximately monthly CTD measurements in the western Channel (intermittent solid line). Key: red diamonds, dashed line, Eastern Channel; red circles, solid line, Celtic Sea; blue circles, solid line, Western Channel Coastal; blue diamonds, dashed line, Western Channel Offshore.

**Figure 4:**
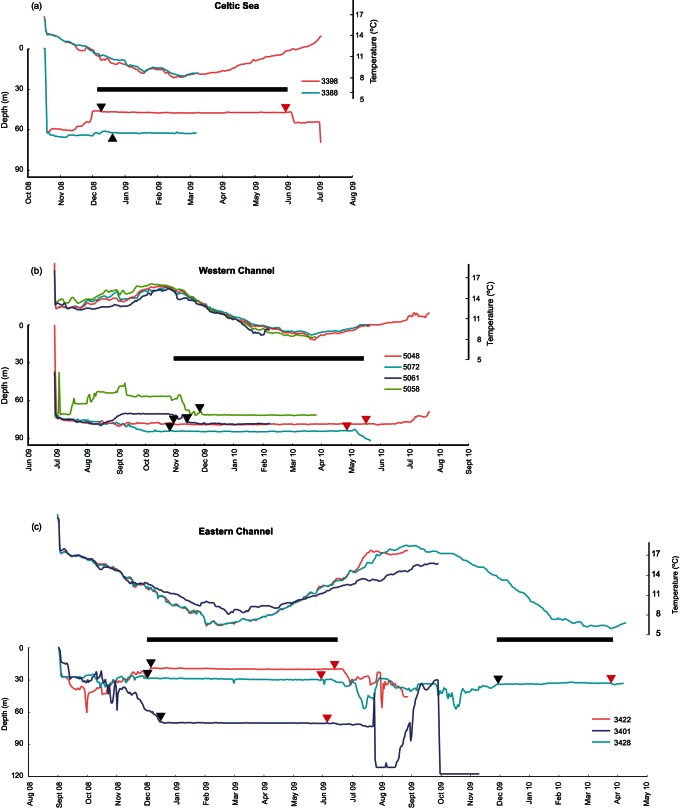
Seasonal depth and temperature experience of individual crabs. Individual temperature (upper plot) and depth (lower plot) records for edible crabs tagged with electronic data storage tags and released in the a) Celtic Sea, b) Western Channel and c) Eastern Channel. Black triangles indicate the onset of “incubation-like” behaviour, red triangles indicate the end of “incubation-like” behaviour. The approximate duration of the egg-incubation period in each area is indicated by a black bar.

Individual variability in depth occupancy was low for CS and western Channel crabs (Figure 3A). By far the greatest levels of depth variation were associated with the EC crabs. Much of this variation was attributable to 3 individuals that recorded data for a year or more, and migrated between 173 and 302 km from the eastern Channel into the western Channel (see below). Individual depth records clearly demonstrate that crabs did not follow defined isobaths (depth contours) during migration (Figure 4).

The seasonal temperature cycle recorded by EC crabs was not fully mirrored by WCC and WCO crabs (Figure 3B *)*. The western Channel crabs, located in deeper, colder, stratified water, experienced rising water temperatures at the start of autumn (i.e. Aug-Oct) 2009, when EC crabs in the previous year had experienced falling temperatures. This period also corresponds with the time when the crabs are most active in terms of migration (see below). However, a comparison with monitored temperatures in the eastern and western Channel suggests that temperatures recorded by the crabs reflect area-specific seasonal trends. The difference between the temperatures recorded by EC crabs and the coastal EC data was commensurate with westward movement in deeper water, further offshore (Figure 1, Figure 3B *)*. Winter (October-January) temperature regimes were very similar for all areas (15°C declining to 9/10°C in January).

### 4. Annual Cycles and Egg Incubation

Six crabs recorded data over a full annual cycle: DST’s 3401, 3422, 3428 (EC), 3398 (CS), 5048 and 5072 (WCO). A further 3 crabs recorded some of the egg-incubation period: DST’s 3388 (CS), 5058 and 5061 (WCC). Individual plots of depth and temperature experience for these crabs are shown in Figure 4, and metrics on the timing and duration of egg incubation are given in Table 2.

**Table 2:** Metrics of egg incubation.

	Tag	Brooding period			Depth		Temperature		Brooding location	
Location		Start	Stop	Duration	Start	Stop	Start	Stop	Lat	Lon
EC	3401	12/12/2008	05/06/2009	175	70.29	71.14	11.45	12.12	49.92	−3.20
	3422	29/11/2008	11/06/2009	194	19.07	19.97	11.8	13.45	50.72	−0.48
	3428	25/11/2008	01/06/2009	188	29.03	30.34	12.1	12.69	50.70	−0.34
	3428	18/11/2009	24/03/2010	126	34.22	33.63	13.44	6.76	50.5	−2.42
CS	3388	16/12/2008	Tag fail		62.17		10.77		50.53	−5.54
	3398	07/12/2008	31/05/2009	175	46.77	46.97	10.74	12.2	50.61	−5.08
WC	5058	26/11/2009	Tag fail		71.15		12.63		49.97	−4.13
	5061	07/11/2009	Tag fail		77.11		13.74		49.92	−4.53
WCO	5048	30/10/2009	16/05/2010	198	78.64	78.23	14.95	10.05	49.69	−3.79
	5072	23/10/2009	25/04/2010	184	84.28	83.74	14.73	9.54	49.52	−4.17

Time period, depth and temperature at onset and completion of incubation, brooding location (estimated using tidal location method), and release and recapture locations of the eight electronic data storage-tagged edible crabs that recorded over all or part of the egg-brooding season. “Tag fail” indicates where data recording ceased before the end of the brooding period. EC, eastern Channel; WCC, Western Channel Coastal; WCO, Western Channel Offshore; CS, Celtic Sea.

For the three EC crabs that recorded data for a year or more, egg incubation was marked by a dramatic cessation of activity between 25/11/08 and 12/12/08 (Table 2). The crabs remained inactive for 175 to 188 days before active foraging recommenced (all crabs were recaptured from baited pots). A similar pattern was observed in western Channel crabs, although overall changes in depth occupancy were less pronounced (Figure 4). Only five DSTs were recaptured from CS. Both crabs that recorded data into the start of egg incubation (Nov-Dec) demonstrated a move into slightly shallower water prior to the onset of brooding. This was most pronounced for DST 3398, which moves up from 63 m to 48 m (Figure 4).

The average depth at which crabs incubated their eggs was 57±23 m, but ranged from 19 m in the shallower eastern Channel, to 84 m in the deeper Western Channel. Incubation lasted on average 177±24 days. The shortest incubation (or at least “incubation-like” behaviour) observed was 126 days during the second brooding season recorded by DST 3428. Temperature at brooding onset was 13±1.5°C, and 11±2°C when feeding recommenced. The lowest temperatures at the onset of incubation were recorded by CS crabs (10.7°C), and the highest in the deepest (WCO) grounds (15°C). By contrast, brooding appeared to stop earlier, and at lower temperatures in the western Channel (although due to sensor failure, we have no data for WCC). Again, the exception was DST 3428, which was the earliest to cease “brooding-like” behaviour (24/03/10), and at the lowest temperature (6.7°C), during her second year. As individual crabs were not returned, we cannot determine whether the crab was actually carrying eggs.

Occasional minor depth fluctuation (>0.5 m) during the incubation period by several females (Figure 4) suggests that the crabs were not always completely immobile during brooding.

### 5. Geolocation and Determination of Brooding Locations

The total number of TLM geolocations that could be generated from a single track was often limited, with >95% of interrogations during the active, migratory period failing to identify positions based on the tidal data recorded. Consequently, TLM estimates of position between release and recapture were effectively limited to those periods when the crabs were sedentary at the times of expected egg incubation.

Channel crabs were not restricted to a single, clearly defined brooding area. Incubation was effected at various locations throughout the Channel (Figure 1, Table 2). All brooding crabs were recaptured west of their brooding locations (some significantly so, e.g. 3422, Figure 1), suggesting that the same incubation sites are not occupied in successive years. DST 3428, the only individual to record data over two brooding seasons, settled down in two separate brooding locations separated by two degrees of longitude in the two successive years.

## Discussion

Here we have described the results from the first large-scale geographical study of the migratory behaviour of crabs using electronic data storage tags. For the first time we have been able to chart the annual migrations of individual crabs, and describe the timing, physical conditions experienced and duration of egg incubation in *Cancer pagurus*.

### 1. Tag Performance and Geolocation

Tag return rates greatly surpassed expectations, with an overall return rate of 34% (compared with 17% overall in Bennett and Brown’s [33] mark-recapture studies). At 17%, fewest DST’s were returned from CS, which appears to reflect the levels of fishing effort experienced in the different areas (M.T. Smith, unpublished data). Recapture rates of 40% in the heavily fished South Devon fishery, are suggestive of intense levels of exploitation.

The interrupted pattern of tag recapture, was similar to that observed in other edible crab tagging programmes [33], [23], and was related to the reproductive activity of the crabs (see below). The tags proved robust to crab behaviour, with only one crab returned missing its DST, and no loss of information due to abrasion of the tag labels. Data download was not possible from two tags (DST’s 5077 and 5098, both WCO) and sensor failure prior to recapture occurred for a further 6 tags. This negatively impacted on our findings on the annual cycle of behaviour for 5 of these individuals, all located in the western Channel. However an overall data capture rate of 82% compares well with analagous studies of fish behaviour [1]. Several tagged crabs were briefly recaptured soon after the initial release, and re-released. It was noted that the crab fishers widely believe that the true position can accurately be determined from the tag, which is not in fact the case. DST 3401, one of the longest data records and furthest migrating crabs, was also the only individual to move into water deeper than 100 m. Our tags were calibrated for use in depths down to 100 m. In this case the sensor functioned to 112 m (15/07/09), then recorded “112” continuously until the crab moved into marginally shallower water after 20 days. However, a second excursion below 112 m on 18/09/09 days resulted in a terminal failure of the sensor.

Unlike the North Sea, where submarine features such as Dogger Bank have proved informative in the reconstruction of DST-tagged fish migrations (e.g. [36]), the sea-bed relief of the English Channel tends to consist of relatively gentle gradients. An exception to this is the “Hurd Deep”, a deep trench of >100 m formed by melting-ice during the previous ice age [38]. This trench effectively divides the northern and southern Channel midway, and in this case provides a useful “waymarker” in the migration route of DST 3401 (above), consistent with the recapture location. Hurd Deep is often cited as a physical barrier between the movements of fish between the northern and southern quadrants of the western English Channel.

The relatively poor performance of TLM was unexpected, and meant that geolocation-based migration-route reconstruction was not possible. Depth data recorded by the crabs often suggested superficially clean records of the tidal conditions necessary in the calculation of geoposition [36], [37]. However subtle movements on the sea-bed, not clearly identifiable as depth changes, seem to have been sufficient to distort the tidal ranges and times of high water required by the data interrogation process. The tidal dynamics of the English Channel are also relatively complex: two tidal “solutions” (i.e. the same time of high water and tidal range) can often occur within a single degree of latitude (see [39]). Consequently, the spread of TLM “solutions” (not shown) were often parallel to, or concentric with, the area where the physical and recapture data hinted might be the more probable location of the crab, reducing the discriminative power of TLM. We were able, however, to identify the brooding locations of the crabs, when no significant horizontal movement occurred over periods of weeks and months.

A possible solution for geolocating crab tracks may be the application of the HMM model [40], where hidden Markov models are applied, incorporating both tidal location and bathymetry as well as using a Kalman filter to take account of previous and subsequent locations in the track record. This was originally developed to help reconstruct the ground-tracks of migrating demersal (sea-bed dwelling) fish which spend only intermittent periods resting on the seabed (principally cod, [40]). However such an exercise would require re-coding of the model to allow for the subtle movements exhibited by migrating crabs, an exercise outside the scope of the current project.

### 2. Reproductive Cycles and Westward Migration

The major breakthrough in the current study lay in describing the timing, conditions and locations of egg-incubation. Previous observations have been restricted primarily to indirect observations on the occurrence and distribution of egg-bearing crabs, either from landings, scuba surveys or aquarium experiments [41], [42], and histological examination [43]. The onset of brooding, or at least the time at which the crabs ceased most activity, corresponded well with previous observations by Latrouite & Phillipe [42] suggesting a mid-November through to early January onset. Naylor *et al*. [44] observed that egg development in North Sea crabs ceased in late November, at which point the eggs entered a period of diapause. Development resumed in late March, with hatching in late June. The appearance of crab larvae in the plankton demonstrates a latitudinal gradient [45], with peak abundance occurring later at higher latitudes. This peak can occur from as early as March off the French Atlantic coast, to as late as August in the northern North Sea [45]. The earliest brooding onset observed in the current study was from late October (WCO). Indeed, westerly crabs appeared to start brooding slightly earlier than those in the eastern Channel, which tended not to commence brooding until mid- to late November.

Howard [41] observed empty guts sealed with a gelatinous “plug” in the majority of ovigerous females, in which the hepatopancreas condition was described as “poor” in 9/10 crabs. Our crabs became largely inactive throughout egg incubation, becoming re-animated towards the end of the brooding period, when foraging recommenced. Although some crab species may remain active during egg incubation [46], Brown and Bennett [47] suggest that ovigerous female *C. pagurus* are largely quiescent whilst incubating. Ungfors [43] collected ovigerous females in the Kattegat from baited nets deployed in April and May, while diver observations of brooding behaviour in a Norwegian fjord between March and August identified some crabs leaving their brooding pits during May and June, and relocating to boulder refuges in the surrounding area [48]. In both latter studies, the activity described could be interpreted as marking the end of the incubation period. All of our crabs were recaptured in baited pots. It is generally accepted that the absence of egg-bearing females in commercial landings over winter is due to non-feeding during incubation. Certainly, some crabs did appear to show more activity within their brooding pits than others. DST 3428 recorded two successive “brooding” seasons. It is noted that she did not moult between broods (although it is thought that sperm retention by the females can facilitate multiple spawning from a single mating event [49], [50]). “Brooding” commenced at similar times in the first and second years (table 2), but there was some evidence that the crab was more active during the second brooding season (daily activity rhythm), which lasted just 126, as opposed to 188 days. Unfortunately, the crab carcass was not generally recovered in these experiments, so we are unable to confirm that our crabs were carrying eggs throughout.

Brooding locations, estimated from TLM, show that brooding is not restricted to a single, clearly defined brooding ground, but may occur at various locations throughout the English Channel (although these are probably defined by substrate characteristics, [41], and that crabs did not show fidelity to the same brooding sites between years.

Both the results presented here and mark-recapture experiments executed in the 1970s [33]–[35] demonstrated long distance, predominantly westward movements by crabs in the English Channel, particularly mature females. In this, like the previous studies, we are unable to rule out the uneven distribution of fishing effort as having influenced these results. However, the westward migration has previously been interpreted as an example of counter-current spawning behaviour [51], [52]. Pre-spawning migration to spawning grounds located in the western Channel have been thought to allow the hatching of planktonic larvae in prevailing tidal currents that will ultimately facilitate the return of settling larvae to their areas of maternal origin. None of our crabs exhibited west to east migration. It is noted however, that with the possible exception of the CS releases, all crabs that recorded the brooding period were migratory. However recent surveys of larval distribution, and interpretation using hydrodynamic modelling (D. Eaton, unpublished data) have suggested insufficient larval transport rates to return western Channel larvae to spawning areas located in the eastern Channel. Furthermore, genetic studies in the Kattegat-Skagerrak area [50] have provided some evidence of large-scale genetic mixing, but significant genetic variation at relatively local scales. The more wide-spread area from which larvae may originate demonstrated by the current study, may help explain at least some of these apparent discrepancies.

### Conclusion

Results from this study provide a vivid demonstration of how the large-scale application of DST’s in an intensively exploited crab fishery is a highly successful and effective means of gathering biological metrics for direct application in the management and conservation of shellfish fisheries. With significant ongoing expansion of coastal and offshore development (e.g. for gravel extraction, renewable energy installations, etc…), we anticipate that future archival tagging of crabs could not only provide a useful means of monitoring stocks, but may also be used to gauge the site-specific impacts of human activities impacting on crab and other stocks.
